# Smartphone-Based Measures as Indicators of Functional Status in Patients With Advanced Cancer

**DOI:** 10.1001/jamanetworkopen.2025.32488

**Published:** 2025-09-18

**Authors:** Marcin Straczkiewicz, Nancy L. Keating, Stephanie M. Schonholz, Ursula A. Matulonis, Neil Horowitz, Susana Campos, Jukka-Pekka Onnela, Alexi A. Wright

**Affiliations:** 1 Department of Biostatistics, Harvard T.H. Chan School of Public Health, Boston, Massachusetts; 2 Department of Health Care Policy, Harvard Medical School, Boston, Massachusetts; 3 Division of General Internal Medicine, Brigham and Women’s Hospital, Boston, Massachusetts; 4 Department of Medical Oncology, Dana-Farber Cancer Institute, Boston, Massachusetts; 5 Harvard Medical School, Boston, Massachusetts; 6 Department of Obstetrics, Gynecology and Reproductive Biology, Brigham and Women’s Hospital, Boston, Massachusetts

## Abstract

This cohort study examines whether digital measures collected from smartphones could enable the remote monitoring of functional status and quality of life among patients with advanced cancer.

## Introduction

In clinical trials of patients with cancer, remote monitoring of patient-reported outcome measures (PROMs) is associated with improved quality of life, physical function, and survival.^1^ However, implementing symptom-focused PROMs in the clinical practice remains resource intensive and requires frequent patient input and clinician engagement.

In parallel, advances in mobile health technologies have enabled passive monitoring of functional status and mobility without additional cost. Smartphones can continuously collect information about movement and mobility with minimal burden. Although wearable activity monitors have been used to follow patients with cancer and other chronic conditions,^2^ to date, clinical validation of smartphone-based measures of physical functioning in advanced cancer populations has been limited, especially over longer time periods. To address this gap, we examined whether digital measures collected from smartphones could enable the remote monitoring of functional status and quality of life among patients with advanced cancer.

## Methods

This cohort study is part of a pilot study of a mobile health intervention that used the Beiwe platform^3^ to collect passive data on patients’ behavior using their personal smartphones. The study was approved by the Dana-Farber/Harvard Cancer Center institutional review board. All participants provided written informed consent. This study followed the STROBE reporting guidelines for observational cohort studies.

Briefly, we recruited patients with recurrent gynecologic cancers receiving systemic (ie, chemotherapy, immunotherapy, or antiangiogenic) or targeted therapies from Dana-Farber Cancer Institute in Boston, Massachusetts (eFigure in Supplement 1). We collected raw triaxial accelerometer and GPS (Global Positioning System) data from smartphones over 180 days. We computed daily measures of gait (step count and cadence) and mobility (number of important locations visited, home time, and distance traveled) from the accelerometer and GPS data, respectively. The statistical methods underlying these measures were developed and validated previously^4,5^; for example, in this population, step counts were estimated to have a mean error of −67.1 steps per day (or 3.4%), compared with a commercial activity tracker, Fitbit Charge 2.^4^ We also collected quality-of-life measures, including Eastern Cooperative Oncology Group Performance Status (ECOG PS) (range, 0-4, with higher numbers denoting worse levels of functioning), EuroQol-5D-5L (EQ5D) subscales (range, 5-25, with higher numbers denoting more problems), and Patient-Reported Outcomes Measurement Information System Physical Function (PROMIS PF 6b) (range, 6-30, with higher numbers denoting better health) at baseline, 30, 90, and 180 days.

Before analysis, participants’ PROMIS PF 6b scores were converted to T-scores. We fit linear mixed-effect models to examine longitudinal associations between smartphone-based gait and mobility measures and PROMs, including categorical (ie, ECOG PS score and EQ5D subscales) and continuous (PROMIS PF 6b) measures. Models contained a random intercept and slope for each patient to account for the correlation induced by repeated measures within patients over time. To determine whether age mediated these associations, we performed a sensitivity analysis, fitting similar models stratified by age into tertiles. When appropriate, we reported 95% CIs and 2-sided *P* values (α = .05). We collected data between April 2017 and August 2020, and data were analyzed from October 2023 to September 2024. Gait measures were computed in MATLAB release 2022a (MathWorks). Mobility measures were computed in Python version 3.9.14 (Python Software Foundation). Statistical analysis was performed using R version 4.1.2 (R Project for Statistical Computing).

## Results

Among 309 patients screened, 220 were excluded who did not meet eligibility criteria (166 patients), already had a wearable (22 patients), did not have a smartphone (14 patients), declined participation (11 patients), or were excluded for other reasons (7 patients). Overall, 89 patients consented to the study; 4 subsequently withdrew. We included 85 individuals (mean [SD] age, 61.6 [10.3] years; all female) with ovarian (64 patients), endometrial (12 patients), and cervical, vaginal, or vulvar (9 patients) cancers (Table).

**Table.** Baseline Demographic, Clinical, and Smartphone Characteristics

Characteristic	Patients, No. (%) (N = 85)
Demographic characteristics	
Female sex	85 (100.0)
Age, y	
Mean (SD)	61.6 (10.3)
Median (range)	62 (24-79)
Race^a^	
American Indian or Alaska Native	1 (1.2)
Asian	4 (4.7)
Black	7 (8.2)
White	67 (78.8)
Other	6 (7.1)
Ethnicity^a^	
Hispanic	2 (2.4)
Non-Hispanic	83 (97.6)
Marital status	
Married or partnered	54 (63.5)
Divorced or separated	12 (14.1)
Widowed	6 (7.1)
Single	12 (14.1)
Unknown	1 (1.2)
Educational status	
High school or less	11 (12.9)
Associate’s degree or some college	22 (25.9)
Bachelor’s degree	23 (27.1)
Graduate degree	29 (34.1)
Employment status	
Full-time	15 (17.6)
Part-time	11 (12.9)
Not working	54 (63.5)
Unknown	5 (5.9)
Clinical characteristics	
Cancer type	
Ovarian	64 (75.3)
Endometrial	12 (14.1)
Cervical, vaginal, or vulvar	9 (10.6)
Height, cm	
Mean (SD)	160.3 (6.3)
Median (range)	160 (146-182)
Weight, kg	
Mean (SD)	73.6 (16.6)
Median (range)	70 (48-119)
Body mass index^b^	
Mean (SD)	28.6 (6.5)
Median (range)	28 (19-51)
Smartphone operating system	
iOS	65 (76.5)
Android	20 (23.5)

^a^ Race and ethnicity were self-reported by study participants. Categories reported as other include Lebanese, West Indian, and Latina.

^b^ Body mass index is calculated as weight in kilograms divided by height in meters squared.

Participants contributed a mean (SD) of 120.0 (62.8) days of smartphone sensor data. After restricting sensor data to valid days (>16 hours of data per day), 74.1% of participants (63 of 85 patients) had data of sufficient quality to analyze, resulting in a mean (SD) of 69.8 (49.3) days for analysis.

Among gait measures, step counts were significantly associated with quality-of-life measures (Figure). Lower patient-reported ECOG PS corresponded to lower step counts (ECOG 3 vs 0, −1837.9 steps per day; 95% CI, −2688.1 to −1007.7 steps per day; *P* < .001), whereas higher PROMIS PF-6b scores were associated with higher step counts (72.64 steps per day per 1-point increase; 95% CI, 39.83 to 105.56 steps per day; *P* < .001) and cadence (0.13 steps per minute; 95% CI, 0.02 to 0.25 steps per minute; *P* = .03). Although minimal clinically important differences for cadence are not universally defined, a decrease of 1000 steps per day has been associated with a 16% increase in the odds of hospitalization or death in patients with advanced cancer.^6^ Participants with lower ECOG PS were also more likely to visit multiple locations per day, compared with those with worse ECOG scores. In sensitivity analyses, these findings did not appear to be age related (data not shown).

**Figure.**
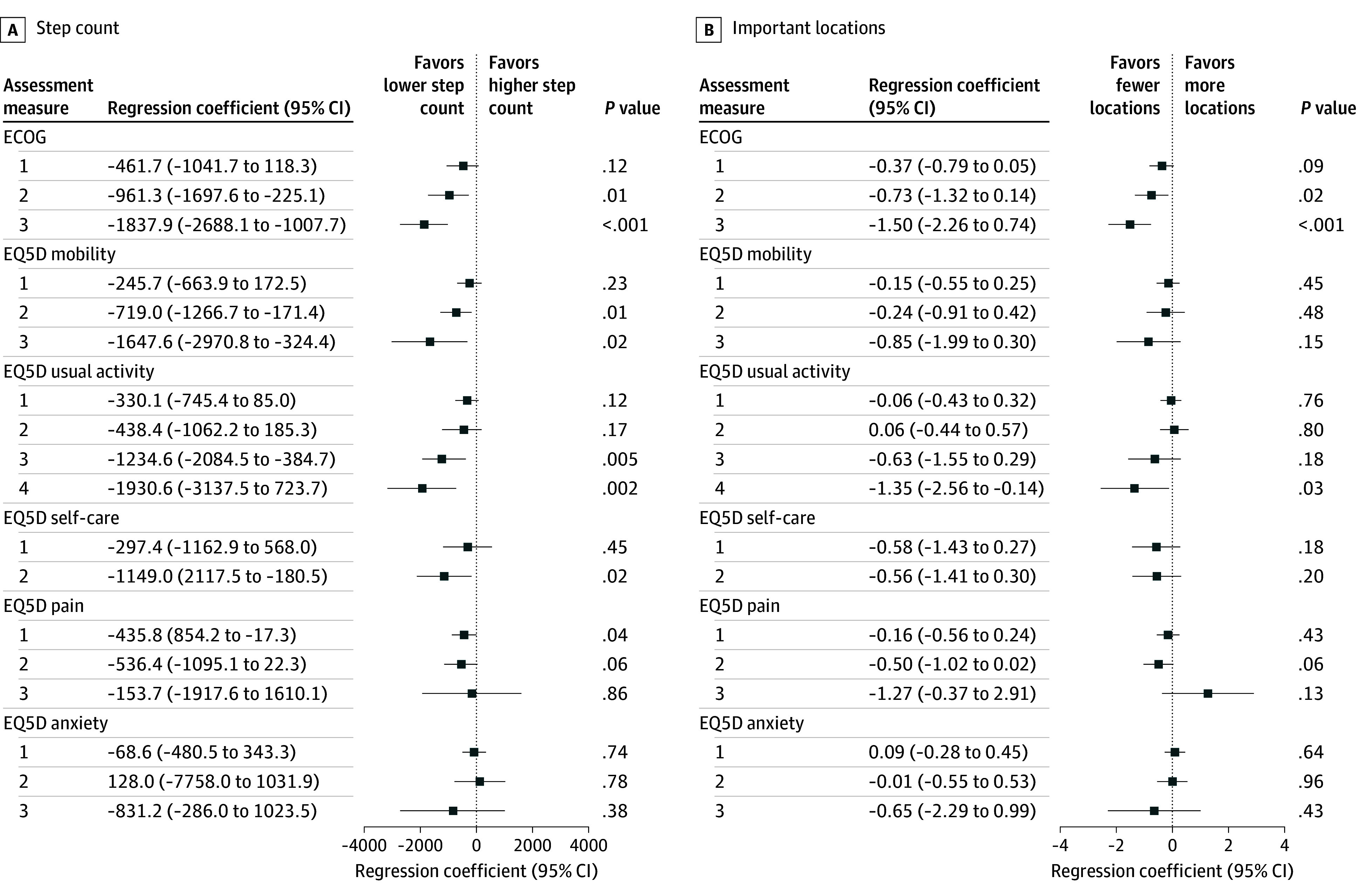
Estimated Regression Coefficients for Eastern Cooperative Oncology Group (ECOG) Performance Status and EuroQol-5D-5L Quality of Life Instrument (EQ5D) Items Forest plots show daily step counts (A), which were estimated from smartphone accelerometer data, and daily number of locations visited (B), which were estimated from smartphone GPS (Global Positioning System) data.

Over the 6-month study period, participants’ walking cadence decreased significantly, although the magnitude of this change was small (−0.23 steps per minute; 95% CI, −0.42 to −0.03 steps per minute; *P* = .02). We did not observe statistically significant longitudinal changes in step counts or other measures of mobility.

## Discussion

This cohort study found that digital measures collected using personal smartphones were associated longitudinally with performance status, quality of life, and physical functioning among patients with advanced cancer. Lesser associations with other mobility measures suggest they may capture distinct behavioral dimensions, rather than direct physical impairments. Limitations include the relatively small number of patients with gynecologic cancers from a single institution. Our findings should also be interpreted considering potential confounding factors, such as baseline function and disease severity, which were not adjusted for because of sample size limitations.

Future research should explore how mobility metrics can complement gait measures to provide a holistic view of patient health in larger samples to determine the generalizability of our findings to other cancer types, sexes, and symptoms (eg, peripheral neuropathy) across the cancer continuum. Overall, our findings demonstrate that smartphone-based digital measures may offer robust, empirical insights into individuals’ health statuses, providing a scalable, low-burden alternative to wearable devices.

## Notes

Supplement 1. **eFigure.** CONSORT Diagram

Supplement 2.
Data Sharing Statement
