# Supplementary material for: Smartphone-Based Measures as Indicators of Functional Status in Patients With Advanced Cancer
Source: JAMA Netw Open. 2025 Sep 18;8(9):e2532488. doi: 10.1001/jamanetworkopen.2025.32488 (PMC12447243; doi:10.1001/jamanetworkopen.2025.32488)
Supplement: Supplement 1. — eFigure. CONSORT Diagram [file jamanetwopen-e2532488-s001.pdf]

Supplemental Online Content

Straczkiewicz M, Keating NL, Schonholz SM, et al. Smartphone-based measures as indicators of functional status in patients with advanced cancer. *JAMA Netw Open*. 2025;8(9):e2532488. doi:10.1001/jamanetworkopen.2025.32488

eFigure. CONSORT Diagram

This supplemental material has been provided by the authors to give readers additional information about their work.

© 2025 Straczkiewicz M et al. *JAMA Network Open*.

eFigure. CONSORT Diagram.

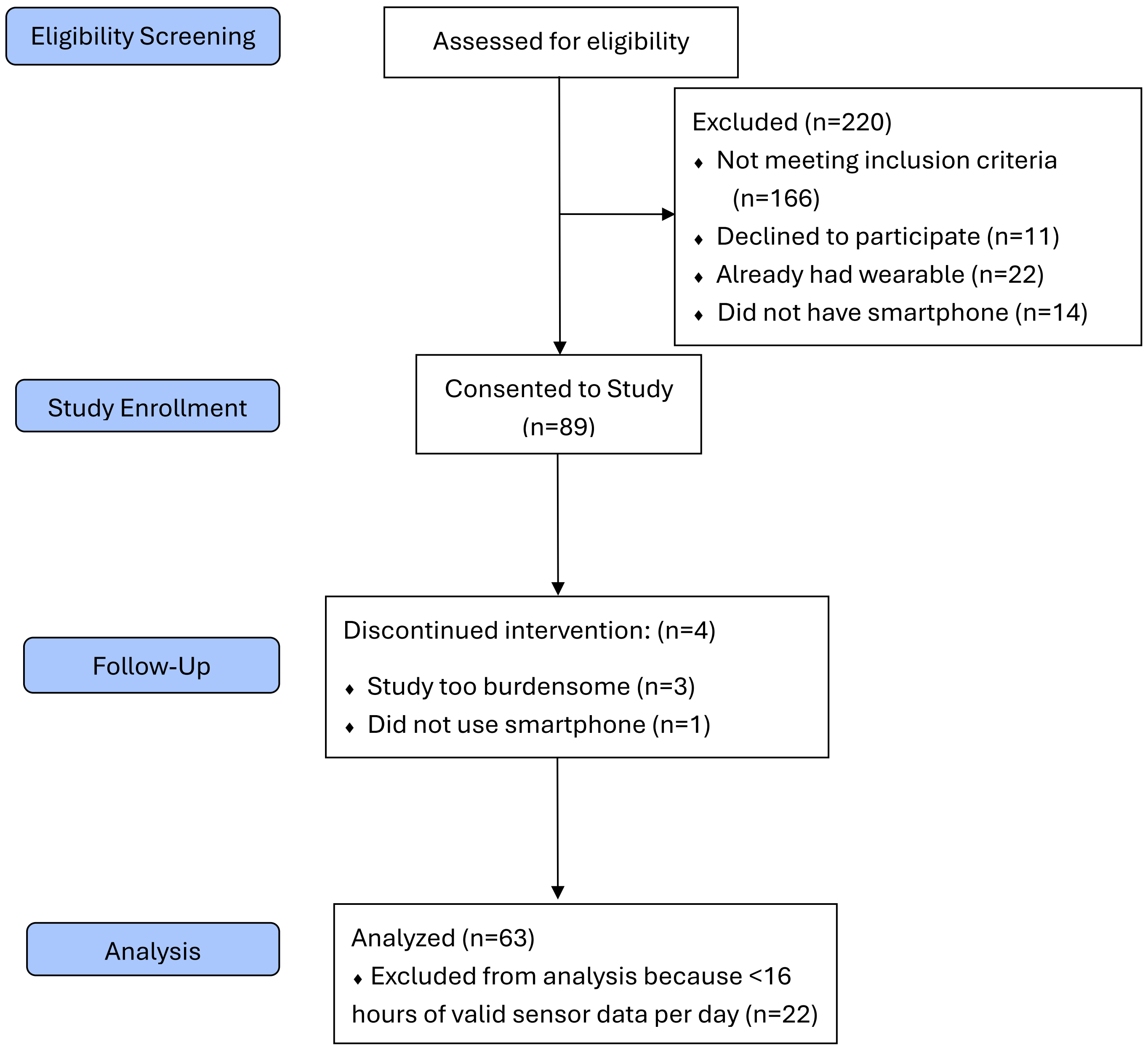

© 2025 Straczkiewicz M et al. *JAMA Network Open*.
