# Supplementary material for: Smartphone-Based Measures as Indicators of Functional Status in Patients With Advanced Cancer
Source: JAMA Netw Open. 2025 Sep 18;8(9):e2532488. doi: 10.1001/jamanetworkopen.2025.32488 (PMC12447243; doi:10.1001/jamanetworkopen.2025.32488)
Supplement: Supplement 2. — Data Sharing Statement [file jamanetwopen-e2532488-s002.pdf]

Data Sharing Statement

Straczkiewicz. Smartphone-Based Measures as Indicators of Functional Status in Patients With Advanced Cancer. *JAMA Netw Open*. Published September 18, 2025. doi:10.1001/jamanetworkopen.2025.32488

Data
Data available: Yes
Data types: Deidentified participant data
How to access data: Deidentified participant data
When available: With publication

Supporting Documents
Document types: Statistical/analytic code
How to access documents: The de-identified data are available upon reasonable request from corresponding author.
When available: With publication

Additional Information
Who can access the data: Researchers whose proposed use of the data has been approved
Types of analyses: For a specified and approved purpose.
Mechanisms of data availability: After approval of a proposal.
